# Awareness and Knowledge of SARS-CoV-2 Infection among Dental Professionals According to the Turkish National Dental Guidelines

**DOI:** 10.3390/ijerph18020442

**Published:** 2021-01-08

**Authors:** Fatih Karayürek, Neslihan Yilmaz Çırakoğlu, Aydin Gülses, Mustafa Ayna

**Affiliations:** 1 Department of Periodontology, Cankiri Karatekin University, Cankiri 18200, Turkey; fatihkarayurek@karatekin.edu.tr; 2 Department of Endodontics, Karabük University, Karabük 78050, Turkey; neslihanyilmazcirakoglu@karabuk.edu.tr; 3 Department of Oral and Maxillofacial Surgery, Universitätsklinikum Schleswig Holstein, Campus Kiel, Christian Albrechts University, 24105 Kiel, Germany; 4 Department of Periodontology, Bonn University, 53111 Bonn, Germany; praxis@ayna-dr.de

**Keywords:** COVID-19, pandemic, dental treatment, Turkish, Asia

## Abstract

The objective of the current survey was to assess the awareness of the dental professionals according to the principals described by the Turkish Dental Association (TDA). A questionnaire including the socio-demographic data, specialties/academic degree, institutions/affiliations, the knowledge about COVID-19, the number of patients examined and dental treatments performed during the COVID-19 pandemic, the knowledge of protection based on the guidelines described by TDA, contamination with COVID-19 and the psychological complaints has been used. A total of 947 (63.1%) dentists enrolled the study. The results showed satisfactory knowledge about the COVID-19 etiology, mode of transmission and the pre-procedural cautions as the majority of them had a fair level of knowledge with significantly higher knowledge among specialized respondents. The participants have also recorded a good judgment towards performing the emergency dental treatment during the current COVID-19 pandemic which corresponds with the guidelines determined by TDA. Considering the updates on the transmission of COVID-19 and protective strategies, there is an urgent need for improvement of dentists’ knowledge about risk assessment via training programs. The incidence of positive testing among dental professionals also necessitates immediately testing of asymptomatic Turkish dental professionals.

## 1. Introduction

The new coronavirus disease (COVID-19) was first identified in China in December 2019. The World Health Organization has now declared that more than 42 million people have been affected, and that more than 1.15 million people have lost their lives. The USA ranks first with approximately 8.8 million people in the number of cases, followed by India (n = 7.8 million) and Brazil (n = 5.3 million) [1].

The first cases were seen in Turkey on 11 March 2020 [2]. After increasing the number of cases with significant momentum until May 2020, Turkey ranks 21st among all the countries in the world with approximately 360,000 cases, whereas nearly 3500 people died (case fatality rate 0.97%) [1]. As of 24 October 2020, despite controversial numbers declared by the Turkish Ministry of Health, Turkey took the 7th place in conjunction with the increasing number of cases in other Asian countries [1]. In October, the case fatality rate in Turkey was about 2.7%, which corresponds to fatality rates in the USA (2.6%) [3].

The dental physicians are one of the most high-risk groups due to their close contact with their patients and dissemination of bio-aerosols during the dental procedure regarding the risk of transmission both for dentists and dental patients [4,5]. Therefore, the dental professionals’ awareness of COVID-19 plays a key role in avoiding transmission.

The most important precautions in a dental practice are; recognizing the risk profile of the patient, prioritizing the most critical dental services and provide care in a way that minimizes harm to patients and personnel from potential exposure to SARS-CoV-2 infection, proactively communicate to both personnel and patients if a symptom exists and know the steps to take if a patient with COVID-19 symptoms enters the dental facility.

To address the above-mentioned precautions, most dental institutions/organizations have defined strict prevention policies and determining strategies for dental management. However, according to the existing literature, dentists’ knowledge of preventive strategies regarding the SARS-CoV-2 infection could vary.

Considering the constantly updated characteristics of COVID-19 and concomitant avoiding strategies, there is a need for studies highlighting the perception to improve the knowledge of dental professionals. Therefore, the aim of the current study was to assess the awareness of the Turkish dental professionals according to the guidelines described by the Turkish Dental Association (TDA).

## 2. Materials and Methods

The study was embarked upon by receiving approval from The Ethics Committee of non-interventional Clinical Research (11 June 2020, 2020/252) and was conducted according to the guidelines of the Helsinki Declaration of Human Rights. All participants were informed and a “Voluntary Informed Consent Form” was obtained from those who accepted to participate in the study. The questionnaire was sent to all dentists in Turkey whose contact information had been provided by email, WhatsApp Messenger (WhatsApp Inc., Menlo Park, CA, USA), Facebook (Facebook, Inc., Menlo Park, CA, USA), and Instagram (Menlo Park, CA, USA). In addition, the questionnaire was also deployed via online forums on social networking platforms.

A questionnaire including the socio-demographic data, specialties (if any)/academic degree, institutions/affiliations, the knowledge about COVID-19, the number of patients examined and dental treatments performed during the COVID-19 pandemic, the knowledge of protection based on the guidelines described by TDA [6], contamination with COVID-19, and the psychological complaints have been sent (Table 1). The study was carried out with the participation of 947 dentists serving in private clinics, universities, and public institutions. The inclusion criteria were being professionally active in the field of dentistry. Obtained data were evaluated with R version 3.2.3. Descriptive analysis (percentage calculations, mean measurements; arithmetic values, minimum values, maximum values), frequency/ratio range were performed. Chi-square tests were used for paired comparisons. In statistical analysis tests, a 95% confidence interval has been applied and the results were considered statistically significant for *p* < 0.05 [7].

**Figure 1:**
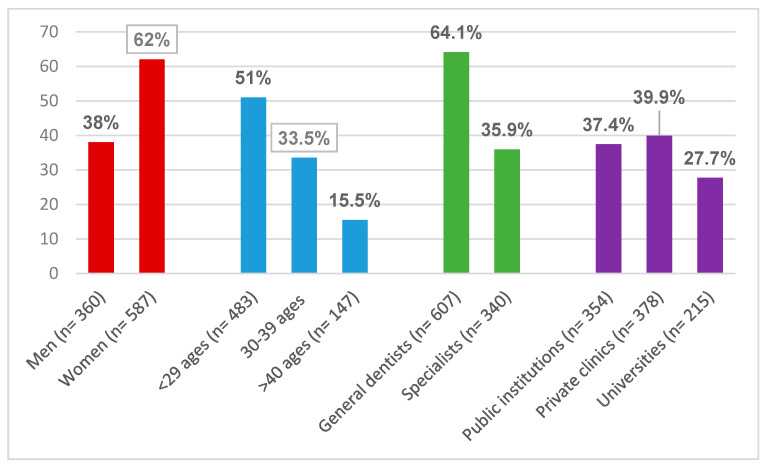
The demographic data of the participants.

Questionnaires were sent to a total of 1500 dental professionals. A total of 947 (63.1%) dentists with an age ranged between 23 and 65 years with a mean age of 31.72 years (62% women and 38% were men) enrolled the study (Figure 1). The mean reaction time to participation was 38.3 h. Regarding dental specialties, 62.6% were general dentists, 10.3% oral surgeons, 6% endodontists, 4.2% pediatric dentists, 3.8% periodontists, 3.4% orthodontists, 3.2% prosthodontists, 2.7% restorative dentistry specialists, and 1.7% were oral diagnosis and radiology specialists. The years of professional experience were as follows: 73.5% between 0 and 10, 17.8% between 11 and 20, and 8.7% were over 21 years of experience. 39.9% of the participants were working in a private hospital/clinic, whereas 7.4% at a public hospital, and 22.7% at university clinics. Among the participants, 12.8% had systemic diseases such as asthma (3%) (n = 28), hypertension (2.7%) (n = 26), heart diseases (1.6%) (n = 15), or diabetes (1.3%) (n = 12).

## 3. Results

The first questions were on the description and symptoms of COVID-19. The open meaning of COVID-19 was responded to correctly by participants (90.1%) and the correct response rate of the classification of COVID-19 was detected as 79.3%.

The majority of participants highlighted high fever as the most common symptom (97.5%). On the other hand, loss of smell and taste was mentioned only by 2.5% of the participants. The most affected system and the country of origin were known by almost all of the participants. (Table 2a).

Participants were asked about the personal protection methods they prefer in their professional and daily lives. The vast majority revealed that they used a surgical mask (96.9%). Regarding the hand disinfection, participants stated that they preferred firstly soap (91.9%), followed by alcohol-based hand disinfectants (68.2%), and 80° Eau de cologne (64.8%).

When the preoperative mouth rinsing percentages were examined, the rate of those who do not apply preoperative antiseptic was 30.4%. Among the preoperative solutions applied, 0.12% chlorhexidine ratio was 23.3%, followed by povidone-iodine solution (20.2%) commercially available mouthwashes with alcohol (16.7%), and oxygenated water (14.6%). There were 30.4% dentists participating in the study (n = 288) who stated that no antiseptic solutions were applied or needed before treatment (Table 2b).

On the other hand, dentists were also asked about the knowledge about which chemical agents could inactivate the virus most effectively. Most of the participants have notified that, viruses lost their efficacy with water–soap (93.1%), and 80° Eau de cologne (84.1%). In addition, 66.2% of participants stated that 70% and above isopropyl alcohol could be also efficient in preventing COVID-19 transmission. Participants were also asked about their preferred protective measures against COVID-19 in their daily life (shopping, public transport, etc.). Wearing a mask and washing hands with soap and water were the most commonly used methods of protection. Hand disinfectants, cologne and surgical gloves have been less commonly preferred protection methods. (Table 2b).

When asked whether the dental treatments can raise COVID-19 infections, the rate of “Yes” response was 40.9% (Table 3). It was remarkable that dentists belonged to the advanced age group who have considered that dental treatments could not increase COVID-19 infection risk (*p* = 0.003) (Table 4). Although the dental examinations by specialist dentists were less than general dentists (*p* = 0.009) during the outbreak, specialist dentists considered that dental treatments could boost COVID-19 infection risk compared to general dentists (*p* = 0.009).

There were 2.5% participants who stated that they have examined at least for once a patient who was COVID-19 positive, whereas 18.3% have answered the question as, "I do not know". Moreover, 1.8% of the participants have confirmed that they were positively tested for COVID-19.

A total of 38% of participants confirmed that there was an isolated room for COVID-19 patients in their institution and 53.7% of them stated that the education presentations against COVID-19 infection were given by intuitional authorities (Table 3).

Participants were asked whether there was an individual with COVID-19 positive diagnosis at their institution. It has been observed that 18.8% and 11.9% of participants answered the question as, "not confirmed but suspected case" and "Yes", respectively. There may be a possibility that "confirmed but suspected case" answers could be particularly explained by either unknown results or ongoing testing processes. Considering the existence of COVID-19 diagnosis in their acquaintances, it was found that 4% said “I do not know”, 5.9% said “Yes” and 90.1% of the participants said “No” (Table 3). The rates of anxiety and fear due to the COVID-19 pandemic was also significantly higher among 358 professionals under 30 years of age (0.000). The need for seeking professional help stress relief of dentists decreased also inversely proportional to age due to pandemic (0.005) (Table 4).

However, the results have clearly revealed that, apart from the institution, most of the dentists had increased anxiety and fear due to the COVID-19 pandemic. Hundred-sixty-one dentists working at the universities (74.9%) showed elevated anxiety and fear levels followed by 241 dentists at the public institutions (68.1%), and 244 dentists working at private clinics (64.6%). The differences among these institutions were also statistically significant (0.034) (Table 4).

Despite highest prevalence in terms of anxiety and fear among dentists working at university clinics, the number of dentists who intend to leave their institutions was found to relatively lower (10.2%). On the other hand, dental professionals working at the private clinics would leave their facility most commonly (24.3%). Concerns about the ongoing academic career might explain the lesser intention to leave the service among dental professionals working at university clinics. Regarding the protection methods, the protective shield was found to be the most preferred equipment (81.3%), followed by surgical mask (81%), surgical gown (75%), protective glass (52.3%), and disposable bonnets (50.1%).

The use of special masks such as N95 (47%), FFP2 (Filtering facepiece) (0.4%), and FFP3 (0.2%) were much lower than traditional surgical masks (Table 2). Comparative analysis regarding the specialization showed that, specialized dentist preferred to use mostly a protective shield (85.6%), followed by surgical glove (81.8%), and surgical mask (78.8%), whereas general dentists preferred mostly surgical mask (82.2%), protective shield (78.9%), and surgical glove (71.2%). It has been observed that protective masks such as N95 were not extensively used in both groups.

The responses of the participants to the list of “applications emerging in dentistry during the COVID-19 epidemic” announced by TDA were also assessed. It was observed that, nearly all dental professionals agreed on the recommended treatment methods. For example, the treatment of the cases such as: “severe toothache caused by pulpal inflammation” (95.2%), “tooth fracture due to trauma” (93.1%), “life-threatening or uncontrolled bleeding” (92.4%), and “jaw and facial fractures” (91.9%) were found to be answered with a high rate of “Yes” by participants. (Table 5).

The results have also revealed that, dentists gave less “Yes” answer to the management of specific cases which requires “biopsy” (57.3%), “management of the temporomandibular joint luxation” (66.6%), or “feeding plate applications by neonates with cleft lip and palate” (60.1%). It could be estimated that those cases were probably more commonly admitted or referred to university clinics, thus their management requires specialization. (Table 6).

There were some differences (in percentage) between general and specialist dentists considering their responses to management of dental emergencies announced by the TDA. For example, it was observed that 72.3% of general dentists mostly performed the management “severe toothache caused by pulpal inflammation”, but this rate remained at 44.7% by specialists. In addition, “abscess or bacterial infection leading to localized pain and swelling” was one of the mostly approved treatment, which was applied by 59.3% of general dentists, the same rate was 40.3% for specialists. Similarly, “severe pain from pericoronitis or third molar” was managed by almost half of the general dentists, this rate was 31.2% among specialists. This difference between general dentists and specialists could be attributed to the fact that, specialists perform only those treatments according to their fields of specialization.

The results of the current study have also showed that, three treatments were common by public institutions and private clinics. It was determined that, the management of “severe toothache caused by pulpal inflammation” was performed at a rate of 75.3% by dentists working in private clinics, whereas this rate was 63% for dentists working at public institutions. The other most commonly applied treatment was the management of "abscess or bacterial infection leading to localized pain and swelling”, with a rate of 52.1% among dentists working at private clinics and 62.7% among those, who work at public institutions. Similarly, “severe pain from pericoronitis or third molar”, was managed by almost half of the dentists working at private clinics (50.3%), this rate was 46.9% for dentists working at public institutions. The results among dentists working at university clinics were diversified, which could be explained by the fact that, the applied treatment could vary from the field of the specialization. (Table 7).

## 4. Discussion

It is very well known that, considering the highly infectious nature of SARS Cov-2, awareness of the symptoms, determining the risk profile for both the patients and the dental team, workflow by high-risk patients, and knowledge of the protective measurements are essential to carry out safe practice to prevent the spread of the disease. These protective measures were determined as wearing FFPE masks, sterilized gloves, and respirators [5]. Considering the results of the current study, it has been observed that most of the Turkish dental professionals used the protective equipment; however, the percentage of carrying FFP-2 masks was low. This could be attributed to the high costs of this equipment. Therefore, critical equipment should be provided by professional organizations such as TDA or Ministry of Health.

The present survey has also shown that the majority of the respondents had a fair level of knowledge, which was significantly more among specialized dental professionals. This percentage is in accordance with the results described by Arora et al. [8], who have evaluated the knowledge of Indian dental professionals. However, in the literature, there are also studies reporting extremely higher levels of knowledge [9]. This difference could be attributed to the diversities regarding the perception and education of dentists among developed and developing countries, which also indirectly defines the position of Turkey.

The results regarding the comparison of knowledge among specialists and practitioners described herein were also similar to the study performed by Quadri et al. [10] in which specialists presented higher knowledge compared to practitioners. Our results also showed no significant differences regarding the distribution of level of knowledge across various age groups and duration of practice. It was also shown that, the participants were mostly familiar with the symptoms of the condition. However, surprisingly, the knowledge about disturbances in smell and taste was insufficient (2.5%). Regarding the mode of transmission and affected systems, nearly all participants had fair knowledge.

Nearly 30% of the respondents, especially those who work at private clinics, perceived COVID-19 as a risk factor during dental treatment. This might be attributed to the fact that, Turkish dentists’ knowledge about the asymptomatic cases, which pose the highest risk due to their act as carriers of infection to others and reservoirs of the disease is unfortunately insufficient. The literature survey revealed higher percentages of knowledge regarding the risk perception among dental professionals [8,11,12,13].

More than 90% of the respondents have performed the emergency dental treatments according to the guidelines described in TDA such as management of pulpitis, abscess, bleeding, etc. However, the need for a second visit to the dental practice for removing stitches might be avoided by using the resorbable sutures.

The majority (66.2%) of the dentists have agreed that, alcohol-based hand disinfectants can be effective in preventing transmission of COVID-19 which was in accordance with the results of the previous studies [14]. In addition, the knowledge of pre-procedural rinsing among Turkish dental professionals was nearly by 70%. It is well known that pre-procedural rinsing should be done with 0.2% povidone-iodine or 0.5–1% hydrogen peroxide; thus, antimicrobial mouth rinses can reduce the load of virus in the oral cavity [15,16,17]. The use of pre-procedural rinsing with 0.2% povidone-iodine or 0.5–1% hydrogen peroxide was performed by nearly 40% of the participants.

The role of dental professionals during a pandemic is another point to discuss. For example, in the last decade, swine flu was the major implication of dental professionals in global health issues [18]. Therefore, in addition to the protective measures regarding the avoidance of the transmission, the dental professionals’ clinical competencies such as medical interview, clinical examination of the head and neck region, swabbing, vitals monitoring, emergency procedures (oxygen-therapy and cardiopulmonary resuscitation) when necessary and basic nursing such as performing infusions, injections or vaccination could be also used during the COVID-19 pandemic. It has been also proclaimed that, in a possible scenario of pandemic such as swine flu, screening the patients for signs of infection and postpone unnecessary dental practice in highly suspected cases might be a good preventive strategy [19]. During the current pandemic, Turkish dental professionals working at public institutions were also commonly assigned to make home testing by contact persons.

The current survey revealed that, young dentists, especially those working at the public facilities of Turkish Ministry of Health suffer relatively higher from anxiety. This could be attributed to the employment of those dentists for controlling and home-swabbing of positively tested COVID-19 cases. On the other hand, it was observed that, anxiety and fear perceived by dentists working at universities were the highest. This might be explained by the fact that, a possible transmission could negatively affect their academical education and professional experience. In addition, despite lower fear and anxiety rates compared to dentists working at university clinics and public institutions, the thoughts about leaving the practice were higher among dentists working at private clinics. This might be explained particularly by “stay at home” campaigns, which resulted in financial concerns.

In the current study, the questionnaire was based on the dental clinical guidelines described by the Turkish Dental Association during the COVID-19 outbreak [6]. A recent research has also focused on the clinical attitudes and behaviors of Turkish dental professionals towards the COVID-19 pandemic, in which an on-line questionnaire was performed with the participation of 1958 dentists [20]. The authors of the study declared that the questionnaire was sent to the participants between 16 March to 20 March 2020. However, the clinical guidelines regarding the COVID-19 outbreak were announced first on 21 March 2020 by the Turkish Dental Association. According to our opinion, the assessment of knowledge of preventative measures should be in accordance with the guidelines described by professional organizations.

Another point that should be mentioned was the increasing number of dental professional, those lost their lives during COVID-19 pandemic. According to the official data provided by the TDA, nine Turkish dental professionals have lost their lives due to COVID-19 [21]. The current survey has also shown that, 1.8% (n = 17) of the participants were also positively tested against COVID-19 which highlights the elevated risk for dental professionals.

In the current study, it could be proclaimed that the participance rate of the dental professionals was relatively high, compared to the previous surveys [22]. This might be explained by the fact that the survey has coincided with the second wave of the COVID-19 pandemic in Turkey and the dentists were highly motivated to participate.

Due to the various oral symptoms of COVID-19 [23], cooperation between dentists and family-and or internal medicine physicians would aid to implement effective early recognition strategies and gain insight in the multidisciplinary management of the condition. Moreover, hospitals around the world implement various measures to prevent the spread of infection among medical staff. Some of the departments at university clinics have also decided to work in double teams to ensure that only one team had a patient contact and the remaining half of the staff could be protected from COVID-19 infection [24].

After “stay home” campaigns, the suspension of management of the patients by dental practitioners has resulted to an increase in the number of the patients admitting the emergency services at various hospitals and/or university clinics. Considering the previously mentioned preventive measures which might cause inadequate number of active medical staff and overload of university clinics, there is a growing need for coordination between dental emergency services at university clinics.

It is well known that; infections of the oral and maxillofacial region include a wide range of conditions from localized dentoalveolar abscesses to deep-neck space infections or more severe cases of necrotizing fasciitis. Therefore, a dental practitioner, who is also responsible for the first-line dental emergency care, needs to be prepared to deal with any infection- related emergencies involving the oral and maxillofacial region. Moreover, the conditions which require inpatient treatment such as; intravenous antimicrobial therapy, extra-oral drainage and/or the patients with existing co-morbidities should be correctly determined and if necessary, the patient should be quickly referred to a hospital with an oral and maxillofacial surgery department or oral and maxillofacial surgery consultant. During the COVID-19 pandemic, there is a need of early and proper diagnosis and if possible, management maxillofacial infections by dental practitioners to avert patients from emergency services, to avoid hospital admissions and to reduce the workload of university clinics [25]. It is crucial that the dental practitioner has knowledge of management of patients with systemic diseases, anatomic boundaries and fascial spaces, identifying the source of the infection, surgical options, administration of the appropriate antimicrobial therapy, and referral to an appropriate provider if indicated [26]. Therefore, the knowledge of dental practitioners about oral and maxillofacial infections should be immediately refreshed and/or expended.

Nowadays, many professional learning activities could/should be performed by using distance-based courses. Accordingly, regional webinars might be helpful in expanding the knowledge of dental practitioners and ensure the cooperation among first- and second line emergency dental care providers during the pandemic.

## 5. Conclusions

In the present study, Turkish dental professionals have revealed satisfactory knowledge about the COVID-19 etiology, mode of transmission and the pre-procedural cautions as the majority of those had a fair level of knowledge with significantly higher knowledge among specialized respondents. The participants have also recorded a good judgment towards performing the emergency dental treatment during the current COVID-19 pandemic which corresponds with the guidelines determined by TDA. Despite having a satisfactory level of knowledge and risk perception, dental practitioners in Turkey declared elevated anxiety and stress levels. Considering the updates on the transmission of COVID-19 and protective strategies, there is an urgent need for improvement of Turkish dentists’ knowledge about risk assessment via training programs. The incidence of positive testing among dental professionals necessitates immediately testing of asymptomatic dental professionals in Turkey.

## Figures and Tables

**Table 1:** Questionnaire.

Age Gender Marital Status Specialty (If any) Experience Duration Institutions COVID-19 Open meaning? Classification of virus? The most obvious symptom? Affected system? The country of origin? When did you quit the dental examination after COVID-19 outbreak? Before and after COVID-19 outbreak, the number of patients you examine? (per day) Which of the following are the emergency practices and treatments you apply in dentistry announced by the Turkish Dental Association regarding the COVID-19 outbreak?
▪ Severe toothache caused by pulpal inflammation ▪ Severe pain from pericoronitis or third molar ▪ Postoperative osteitis or alveolitis ▪ Abscess or bacterial infection leading to localized pain and swelling ▪ Tooth fracture causing pain or soft tissue trauma ▪ Tooth fracture due to trauma ▪ Jaw and facial fractures ▪ Acute and painful lesions/ulcerations of the oral mucosa ▪ Life-threatening or uncontrolled bleeding ▪ Intraoral/extraoral infections that threaten the patient’s breathing ▪ Treatment of patients undergoing radiotherapy or chemotherapy treatment ▪ Patients seeking dental consultation for medical problems ▪ Removing stitches ▪ Treatment of temporary restoration loss/fractures and traumatic mucosal ulcerations that prevent the use of removable prostheses without creating aerosol ▪ Pain and/or infection due to injury to the soft tissue caused by breaking the brackets or dental braces of patients under orthodontic treatment ▪ Feeding plate applications of newborn patients with cleft lip and palate ▪ Luxation of temporomandibular joint ▪ Biopsy
15. Protective measurements in dental examinations? 16. Antiseptic Solutions before dental examination? 17. Protective measurements in daily life? 18. Chemical Agents to prevent virus? 19. Have you had symptoms of COVID-19? Have you been diagnosed with COVID-19? 20. Has any of your family, close relatives or neighbors been diagnosed with COVID-19? 21. Has anyone been diagnosed COVID-19 positive in the healthcare institution you work at? 22. Is an isolated room provided for COVID-19 at the institution you work at? 23. Have you applied dental treatment to any patient diagnosed with COVID-19? 24. Education provided by the institution you work at on COVID-19? 25. Thoughts about leaving your profession because you are in the high-risk group after the pandemic? 26. Have you experienced problems causing anxiety or fear due to the COVID-19 pandemic? 27. Did you need for seeking professional help stress relief during the COVID-19 pandemic?

**Table 2:** (**a**) Knowledge regarding COVID-19. (**b**) Knowledge regarding protective measurements.

(**a**)	
	**n** **(%)**
**COVID-19 Open meaning?**	
Yes	853 (90.1)
No	94 (9.9)
**Classification of the virus?**	
MERS-CoV-1	26 (2.7)
MERS-CoV-2	86 (9.1)
SARS-CoV-1	84 (8.9)
SARS-CoV-2	751 (79.3)
**The most common symptom?**	
The loss of smell and taste	24 (2.5)
High Fever	923 (97.5)
**The affected system?**	
The respiratory system	945 (99.8)
The circulatory system	1 (0.1)
The digestive system	1 (0.1)
**The country where it was first identified?**	
China	946 (99.9)
Italy	1 (0.1)
(**b**)	
	**n (%)**	
**Antiseptic solutions before dental examination?**	
0.12% Chlorhexidine	221 (21.3)
Povidone-iodine	191 (20.2)
No solutions	228 (30.4)
Mouthwash	158 (16.7)
Oxygenated water	138 (14.6)
**Protective measurements in daily life?**	
Mask	918 (96.9)
Soap	870 (91.9)
Hand disinfectants	646 (68.2)
80° Eau de cologne	614 (64.8)
Surgical Gloves	461 (48.7)
**Chemical Agents to prevent virus?**	
Water and Soap	882 (93.1)
80° eau de cologne	796 (84.1)
70% and above isopropyl alcohol	627 (66.2)
Antibacterial hand wipes	236 (24.9)
**Protective measurements during dental examinations?**	
Protective shield	770 (81.3)
Surgical mask	767 (81.0)
Surgical gown	710 (75.0)
Protective glass	495 (52.3)
Disposable bonnet	474 (50.1)
N95/FFP2 masks	445 (47.0)

**Table 3:** COVID-19 positivity, facilities and educational status.

	**n (%)**
**Have you had symptoms of COVID-19? Have you been diagnosed with COVID-19?**	
Yes	17 (1.8)
No	930 (98.2)
**Has any of your family, close relatives or neighbors been diagnosed with COVID-19?**	
N/A	38 (4)
Yes	56 (5.9)
No	853 (90.1)
**Increased COVID-19 risk due to the dental treatment?**	
Yes	387 (40.9)
No	560 (59.1)
**Has anyone been diagnosed COVID-19 positive in the healthcare institution you work at?**	
I do not know	178 (18.8)
Yes	113 (11.9)
No	656 (69.3)
**Is an isolated room provided for COVID-19 at the institution you work at?**	
Yes	360 (38)
No	587 (62)
**Have you applied dental treatment to any patient diagnosed with COVID-19?**	
I do not know	173 (18.3)
Yes	24 (2.5)
No	750 (79.2)
**Education provided by the institution you work at on COVID-19?**	
Yes	509 (53.7)
No	438 (46.3)

**Table 4:** Assessment of anxiety and fear.

	<29 Ages Public n (%)	30–39 Ages Private n (%)	>40 Ages University n (%)	*p*
**Increased risk of COVID-19 transmission due to dental treatment?**				
Yes	**220** **(45.5)**	**125** **(39.4)**	**42** **(28.6)**	0.001 *
	171 (48.3)	108 (28.6)	108 (50.2)	0.000 *
No	**263** **(54.5)**	**192** **(60.6)**	**105** **(71.4)**	
	183 (51.7)	270 (71.4)	107 (49.8)	
**Have you applied dental treatment to any patient diagnosed with COVID-19?**				
I do not know	**90** **(18.6)**	**63** **(19.9)**	**20** **(13.6)**	0.030 *
	83 (23.4)	61 (16.1)	29 (13.5)	0.000 *
Yes	**19** **(3.9)**	**3** **(0.9)**	**2** **(1.4)**	
	22 (6.2)	0 (0.0)	2 (0.9)	
No	**374** **(77.4)**	**251** **(79.2)**	**125** **(85)**	
	249 (70.3)	317 (83.9)	184 (85.6)	
**Have you experienced problems causing anxiety or fear due to the COVID-19 pandemic?**				
Yes	**358** **(74.1)**	**205** **(64.7)**	**83** **(56.5)**	0.000 *
	241 (68.1)	244 (64.6)	161 (74.9)	0.034 *
No	**125** **(25.9)**	**112** **(35.3)**	**64** **(43.5)**	
	113 (31.9)	134 (35.4)	54 (25.1)	
**Did you need for seeking professional help for stress relief during the COVID-19 pandemic?**				
Yes	**89** **(18.4)**	**42** **(13.2)**	**12** **(8.2)**	0.005 *
No	**394** **(81.6)**	**275** **(86.8)**	**135** **(91.8)**	

Statistically significant differences were marked with *. Bold numbers show the distribution regarding the age group of the participants.

**Table 5:** The answer rate of participants to the list of emergency practices and treatments declared by Turkish Dental Association (TDA) and protective measurements.

	General Dentists n (%)	Specialists n (%)
**Which of the following are the emergency practices and treatments you apply in dentistry announced by the Turkish Dental Association regarding the COVID-19 outbreak?**		
Tooth fracture causing pain or soft tissue trauma	95 (15.7)	63 (18.5)
Removing stitches	258 (42.5)	101 (29.7)
Temporary restorations without creating aerosol	225 (37.1)	42 (12.4)
Abscess infection leading to localized pain and swelling	360 (59.3)	137 (40.3)
Acute and painful lesions/ulcerations of the oral mucosa	74 (12.2)	26 (7.6)
Severe pain from pericoronitis or third molar	308 (50.7)	106 (31.2)
Severe toothache caused by pulpal inflammation	439 (72.3)	152 (44.7)
**Protective measurements during dental examinations**		
Surgical mask	499 (82.2)	268 (78.8)
Disposable bonnet	326 (53.7)	206 (60.6)
Bonnet	177 (29.2)	82 (24.1)
Surgical gown	432 (71.2)	278 (81.8)
Protective glass	328 (54)	167 (49.1)
Protective shield	479 (78.9)	291 (85.6)
N95 mask	279 (46)	166 (48.8)

**Table 6:** The responses of the participants according to the treatment list declared by the TDA.

	n (%)
**The number of applications in the list of emergency treatments announced by TDA in dental clinics**	
Severe toothache caused by pulpal inflammation	902 (95.2)
Tooth fracture due to trauma	882 (93.1)
Life-threatening or uncontrolled bleeding	875 (92.4)
Jaw and facial fractures	870 (91.9)
Intraoral/extraoral infections that threaten the patient’s breathing	846 (89.3)
Abscess or bacterial infection leading to localized pain and swelling	825 (87.1)
Tooth fracture causing pain or soft tissue trauma	814 (86)
Severe pain from pericoronitis or third molar	804 (84.9)
Postoperative osteitis or alveolitis	787 (83.1)
Pain and/or infection due to injury to the soft tissue caused by breaking the brackets or dental braces of patients under orthodontic treatment	734 (77.5)
Treatment of patients undergoing radiotherapy or chemotherapy treatment	717 (75.7)
Treatment of temporary restoration loss/fractures and traumatic mucosal ulcerations that prevent the use of removable prostheses without creating aerosol	689 (72.8)
Acute and painful lesions/ulcerations of the oral mucosa	671 (70.9)
Removing stitches	656 (69.3)
Luxation of temporomandibular joint	634 (66.9)
Patients seeking dental consultation for medical problems	577 (60.9)
Feeding plate applications of newborn patients with cleft lip and palate	569 (60.1)
Biopsy	543 (57.3)

**Table 7:** The treatments applied by the participants in their clinics according to the treatment list declared by the TDA.

	Private Clinic n (%)	Public Institution n (%)	University n (%)	*p*
**The number of applications in the list of emergency treatments announced by TDA in dental clinics**				
Tooth fracture causing pain or soft tissue trauma	63 (16.7)	55 (15.5)	40 (18.6)	0.000 *
Removing stitches	154 (40.7)	144 (40.7)	61 (28.4)	
Temporary restorations without creating aerosol	124 (32.8)	126 (35.6)	17 (7.9)	
Abscess/bacterial infection leading to localized pain and swelling	197 (52.1)	222 (62.7)	78 (36.3)	
Acute and painful lesions/ulcerations of the oral mucosa	32 (8.5)	53 (15)	15 (7)	
Severe pain from pericoronitis or third molar	190 (50.3)	166 (46.9)	58 (27)	
Severe toothache caused by pulpal inflammation	285 (75.4)	223 (63)	83 (38.6)	

Statistically significant differences were marked with *.

## Data Availability

Not applicable.
